# Regional Evidence on Neonatal Abstinence Syndrome: Association Between Finnegan Score Severity and Laboratory Findings in a Tertiary NICU

**DOI:** 10.3390/healthcare14121639

**Published:** 2026-06-10

**Authors:** Melda Tas Gungor, Dilek Kahvecioglu

**Affiliations:** Department of Neonatology, University of Health Sciences, Ankara Training and Research Hospital, Ankara 06200, Türkiye; dileksaracoglu@yahoo.com

**Keywords:** neonatal abstinence syndrome, withdrawal severity, Finnegan score, liver enzymes, aspartate aminotransferase, alanine aminotransferase, neonatal intensive care unit, Turkey

## Abstract

**Highlights:**

**What are the main findings?**

- Higher Finnegan scores were associated with higher white blood cell counts and liver enzyme levels (AST and ALT), with moderate-to-large effect sizes, in neonates exposed to maternal substance use.
- Infants placed under state care had significantly longer hospital stays, highlighting the impact of social factors in a region with limited data on neonatal abstinence syndrome. These preliminary findings require confirmation in larger cohorts.

**What are the implications of the main findings?**

- Severe neonatal withdrawal may reflect not only neurological symptoms but also systemic involvement, including potential hepatic effects, even in underrepresented populations.
- Sociocultural factors and potential underreporting in regions with limited data may influence both clinical recognition and healthcare burden of NAS, emphasizing the need for region-specific research.

**Abstract:**

**Objective**: Maternal substance use during pregnancy is an increasing public health concern worldwide. However, data on neonatal abstinence syndrome (NAS) remain limited in many regions, including Turkey. This study aimed to evaluate the clinical and laboratory characteristics of neonates exposed to maternal substance use and to assess the association between Finnegan score severity and laboratory findings. **Methods**: This retrospective study was conducted in a tertiary neonatal intensive care unit. Neonates with prenatal substance exposure were divided into two groups according to their Modified Finnegan scores (<8 and ≥8). Demographic characteristics, clinical outcomes, and laboratory parameters, including acute phase reactants and liver enzymes, were compared between the groups. Maternal substance exposure was mainly determined by maternal self-report, with toxicological confirmation available in only two infants. **Results**: A total of 25 neonates were included. Higher Finnegan scores were associated with increased white blood cell counts, plateletcrit and liver enzyme levels (AST and ALT), although only the ALT association remained significant after correction for multiple comparisons. Infants placed under state care had longer hospital stays than those discharged to their families (*p* = 0.02). No mortality was observed. **Conclusions:** In this small retrospective cohort, greater withdrawal severity was associated with higher aminotransferase levels, particularly ALT, and longer hospitalization was observed among infants placed under state care. These findings should be regarded as preliminary and require confirmation in larger prospective multicenter studies.

## 1. Introduction

Substance and drug use during pregnancy has become an increasingly important global public health concern. The most commonly used substances in this period are alcohol, tobacco and cannabis. In the United States, there was a fourfold increase in the rate of opioid use disorder documented at the time of delivery or admission to hospital between 1999 and 2014 [1]. Substance use among pregnant women has increased over recent decades, affecting a substantial number of newborns each year; therefore, education and counseling of women of reproductive age are important preventive strategies [2]. This reflects an expanding burden of prenatal substance exposure on maternal and neonatal health systems. Prenatal substance exposure has been associated with an increased risk of adverse perinatal outcomes, including miscarriage, preterm birth, and fetal growth restriction, as well as long-term neurodevelopmental and behavioral disorders such as attention deficit and cognitive impairment in offspring [3,4,5]. Furthermore, it is hypothesized that the risk of depression and anxiety increases in these children [3].

The condition affecting infants after birth due to the sudden withdrawal of substances to which the fetus was exposed is known as Neonatal Abstinence Syndrome (NAS). This condition is a syndrome characterized by a range of symptoms that manifest in infants exposed to certain substances in utero, most commonly opioids [6]. A significant escalation in hospital expenses has been observed, attributable to the treatment of female and neonatal opioid-dependent patients who were compelled to be hospitalized for a protracted period, often spanning several weeks, to address withdrawal symptoms [3]. The Finnegan Score is utilized as a standardized assessment tool to identify and monitor newborns with documented in utero opioid exposure or who have shown high likelihood of exposure in tests. The assessment tool is designed to evaluate the severity of withdrawal symptoms, which may include tremors, irritability, excessive crying and feeding difficulties. It assigns a score to each symptom to provide a comprehensive assessment of the individual’s condition. The presence of a Finnegan score > 8, although not specifically mentioned during pregnancy, is an indication of opioid exposure [6]. The severity of NAS is evaluated using the Finnegan scoring system and classified as absent, mild and severe [7].

Although NAS is mainly considered a neurological and behavioral withdrawal syndrome, prenatal substance exposure may also affect other organ systems. Some hypotheses have been developed that NAS increases oxidative stress and cytokine levels and triggers inflammation, but the exact mechanism has not yet been elucidated [8,9,10,11]. A range of substances associated with NAS, including alcohol and opioids, are metabolized in the liver and pose a risk of potential liver damage in newborns. However, data directly examining the relationship between withdrawal severity and neonatal liver enzyme abnormalities remain limited.

Despite the increasing global burden of neonatal abstinence syndrome, most available data originate from North America and Western Europe, while clinical data from regions such as Turkey remain limited. Social stigma, limited disclosure, and the absence of systematic perinatal substance-use surveillance may contribute to underrecognition of maternal substance use, restricting the generalizability of existing evidence. Consequently, region-specific studies are needed to better characterize NAS in underrepresented populations. This study aimed to evaluate whether Finnegan score severity was associated with laboratory parameters, particularly aminotransferase levels and selected hematological indices, as potential markers of systemic or hepatic involvement.

## 2. Materials and Methods

This retrospective observational study was conducted in a tertiary neonatal intensive care unit (NICU). Medical records of neonates admitted between January 2018 and January 2023 due to maternal substance use were reviewed. All neonates admitted to the NICU because of documented maternal substance use during pregnancy were eligible for inclusion. Maternal substance exposure was primarily determined by maternal self-report obtained during routine clinical assessment. Toxicological confirmation was not routinely performed and was available in only two infants. The complete data were available for all included patients. Demographic, maternal, clinical, and laboratory data were extracted retrospectively from hospital electronic records and patient charts. The following variables were recorded:

- Maternal age and substance exposure history;
- Gestational age and birth weight;
- APGAR scores;
- Respiratory and inotropic support requirements;
- Echocardiographic and transfontanel ultrasonographic findings;
- Length of hospital stay;
- Need for social services intervention.

Laboratory parameters included white blood cell count (WBC) (N: 6000–30,000/mm^3^), platelet count (PLT) (N: 150,000–400,000/mm^3^), mean platelet volume (MPV), plateletcrit (PCT) (%), and neutrophil-to-lymphocyte ratio (NLR) (N: 0.52–0.91). Levels of aspartate aminotransferase (AST) (U/L) and alanine aminotransferase (ALT) (U/L) from liver function tests; creatinine (mg/dL) from kidney function tests; and C-reactive protein (CRP) (N: 0–10 mg/L) as an acute-phase reactant were also evaluated. A complete blood count was performed immediately following birth and biochemical blood samples were obtained 24 h later. According to the CALIPER study, the upper reference limits during early infancy are approximately 55 U/L for AST and 23 U/L for ALT, although values may vary according to postnatal age and gestational maturity [12]. The hematological indices were selected as inexpensive, routinely available markers of systemic inflammation and platelet activation. NLR has been established as a reliable marker of systemic inflammation in neonatal conditions including sepsis and SIRS, and platelet indices such as MPV and plateletcrit have similarly been used as accessible biomarkers of the neonatal inflammatory response [13,14,15,16]. These indices were previously reported to be altered in neonates with NAS [17], and we therefore selected them to explore the proposed inflammatory component of withdrawal.

Withdrawal severity was assessed using the Modified Finnegan Neonatal Abstinence Scoring System [6]. Infants were scored every 8 h during hospitalization. Non-pharmacological interventions, including minimization of environmental stimuli, swaddling, and supportive feeding practices, were applied as first-line management. Pharmacological treatment was initiated in infants with two consecutive Finnegan scores > 8 or a single score ≥ 12 [18]. For analytical purposes, infants were categorized according to their highest recorded Finnegan score into a low-severity group (<8) and a high-severity group (≥8). Phenobarbital (5 mg/kg/day) was used as the first-line pharmacological treatment according to unit protocol.

Because maternal substance exposure was primarily based on self-report, misclassification of exposure could not be excluded. To minimize selection bias, all eligible infants admitted during the study period were included consecutively. As this was a retrospective study, no a priori sample size calculation was performed. The study population consisted of all eligible neonates identified during the five-year study period. A comparative analysis was conducted on the demographic data, clinical data and laboratory values of the two groups. The patients were anonymous. The study was approved by the institutional ethics committee.

### Statistical Analysis

Statistical analyses were performed using IBM SPSS Statistics version 22.0 (IBM Corp., Armonk, NY, USA). Data distribution was assessed using the Kolmogorov–Smirnov test. Continuous variables were presented as mean ± standard deviation or median (minimum–maximum), as appropriate. Categorical variables were presented as frequencies and percentages. Between-group comparisons were performed using Student’s *t*-test or Mann–Whitney U test for continuous variables and chi-square test for categorical variables. In addition to *p*-values, effect sizes (Cohen’s d) and 95% confidence intervals were calculated. Because multiple laboratory parameters were evaluated, findings were interpreted as exploratory and hypothesis-generating.

## 3. Results

The demographic, clinical and laboratory features of infants admitted to the NICU due to maternal substance abuse between January 2018 and January 2023 were assessed. During the study period, 3302 patients were admitted to the neonatal intensive care unit. A total of 25 infants, representing 0.76% of NICU admissions during the study period, were diagnosed with NAS. All of the infants in this study were inborn patients. The patient demographics are presented in Table 1, which shows that there was no significant disparity between the two groups. The predominant drug used by mothers was heroin, accounting for nine patients (36%). No significant association was found between heroin use and Finnegan score. Further details on other substances used are provided in Table 2. Two cases of premature rupture of the membranes (PROM) were observed, two were diagnosed with congenital syphilis, two patients tested positive for HBsAg, and one mother tested positive for HIV. One infant was diagnosed with congenital CMV infection, and ganciclovir treatment was administered. One patient was diagnosed with pyloric stenosis and underwent surgical intervention.

Post-delivery, positive pressure ventilation (PPV) was required for three newborns (12%). Of the infants, one exhibited a Finnegan score of <8, and two demonstrated higher scores. The mean highest Finnegan score was 9.3 (minimum 4–maximum 16). Of the 14 infants with Finnegan scores ≥ 8, 13 (92.8%) received phenobarbital (FB) therapy. One patient (7.1%) received additional morphine, while multiple antiepileptic therapies were initiated in another patient. Mechanical ventilation was required in eight patients (32%), five of whom (35.7%) had high Finnegan scores and three of whom (27.3%) had low Finnegan scores. The echocardiogram findings indicated that seven patients (28%) presented with patent foramen ovale, while six patients (24%) had secundum atrial septal defect. One patient exhibited a wide patent ductus arteriosus (4%) and mild tricuspid regurgitation. There was no significant difference in terms of cardiac defects among the study groups. The transfontanel ultrasonography of all infants was normal.

Laboratory parameters are summarized in Table 3. Compared with infants in the low-severity group (Finnegan < 8), those in the high-severity group (Finnegan ≥ 8) showed higher WBC (mean difference 9549/mm^3^, 95% CI 2769–16,329; Cohen’s d = 1.11; *p* = 0.009), higher PCT (mean difference 0.07%, 95% CI −0.01 to 0.15; d = 0.65; *p* = 0.041), and borderline-higher MPV (mean difference 0.50 fL, 95% CI 0.00–1.00; d = 0.78; *p* = 0.050). AST and ALT were also significantly higher in the high-severity group (AST: mean difference 46.2 U/L, 95% CI 14.8–77.6; d = 1.13; *p* = 0.009; ALT: mean difference 21.4 U/L, 95% CI 8.8–34.0; d = 1.31; *p* < 0.001). After Bonferroni correction for the nine biochemical comparisons (α = 0.0056), only the ALT difference remained statistically significant. No statistically significant differences were observed in NLR, CRP, PLT, or creatinine between the groups (Table 3).

Social workers were consulted for all patients. A total of 10 infants (40%) were placed into state care due to either family abandonment or decisions made by social services. The mean duration of stay for all patients was 30.2 days, with a range from a minimum of 7 days to a maximum of 76 days. However, the average length of stay for infants placed in social services was 43 ± 17.2 days, whereas the average length of stay for infants who returned to their families was significantly lower at 24.4 ± 14.6 days (*p* = 0.02). No mortality was observed in any of the patients.

## 4. Discussion

Maternal substance use represents an important and growing challenge for neonatal healthcare systems worldwide. In this study, we evaluated the clinical and laboratory characteristics of infants born to substance-using mothers and examined the association between withdrawal severity and laboratory parameters. Our findings contribute to the limited body of literature on the clinical consequences of prenatal substance exposure in neonatal intensive care units.

The consequences of maternal substance and drug abuse have been demonstrated to persist beyond the early stages of childhood. A substantial body of research has indicated that offspring of substance-abusing parents are more likely to develop a substance use disorder. Furthermore, these individuals are susceptible to internalizing and externalizing issues, including anxiety, depression, and behavioral problems [4]. A further study found that mothers with substance abuse demonstrated reduced reflective functioning, which had a negative impact on their ability to provide sensitive and responsive care [19].

The ALT association, which remained significant after correction for multiple comparisons, represents the most robust laboratory finding in this cohort. In contrast, higher WBC and PCT should be interpreted as secondary, hypothesis-generating observations because they were nominally significant but did not remain significant after multiple-comparison correction. Data directly evaluating transaminase abnormalities in neonates with NAS are scarce, and the available evidence is largely indirect. Several substances represented in our cohort have plausible hepatic relevance through different mechanisms. Alcohol exposure has been associated with multi-organ fetal effects, including hepatic involvement; prenatal cocaine exposure may contribute to placental and fetal ischemia through vasoconstriction [20,21]. In addition, opioids undergo extensive hepatic metabolism [22].

Interpretation of aminotransferase levels during the neonatal period is challenging because normal values are higher and more variable than in older children, particularly among preterm infants [12,23,24,25]. In the study by Badakhshan et al., the upper reference limits were approximately 55 U/L for AST and 23 U/L for ALT [23]. In the present cohort, mean AST and ALT values in the high-withdrawal group exceeded these reference limits. However, aminotransferase elevations may also reflect prematurity, perinatal hypoxic–ischemic stress, sepsis, or hemolysis rather than substance exposure alone. Although our findings are consistent with an association between greater withdrawal severity and higher AST and ALT levels, the small sample size, heterogeneous exposures, and retrospective design preclude causal inference. Moreover, since the proportions of preterm and heroin-exposed infants did not differ significantly between the two Finnegan groups, these factors are unlikely to fully explain the observed differences, although residual confounding cannot be excluded.

In our cohort, infants with higher withdrawal severity had significantly higher white blood cell counts and PCT, with borderline-higher MPV, whereas NLR and CRP did not differ. This pattern is consistent with a previous report describing elevated WBC and MPV in neonates with NAS [17]. It aligns with preclinical evidence that prenatal opioid exposure is accompanied by a pro-inflammatory state, with elevated cytokines such as TNF-α and increased microglial activation in the developing brain [8,10]. A study conducted on adult patients revealed that individuals diagnosed with alcohol, heroin, or synthetic cannabinoid dependence exhibited elevated NLR values in comparison to healthy control groups. Conversely, no statistically significant disparities were observed in CRP levels [26]. In a separate study, it was observed that adult individuals with a dependence on cocaine had elevated levels of the neutrophil-to-lymphocyte ratio (NLR) [27]. Although adult studies suggest that NLR may be altered in substance-use disorders, these data cannot be directly extrapolated to neonates with NAS because neonatal immune responses, perinatal stressors, and developmental physiology differ substantially from those of adults. The absence of differences in NLR and CRP, however, suggests that any inflammatory signal is partial and should be interpreted cautiously given the small sample and multiple comparisons. The dissociation between elevated WBC and the absence of CRP/NLR differences may reflect a transient, non-acute-phase pattern of hematological activation; CRP and NLR are also influenced by perinatal infection and stress, which were not uniformly distributed in our small sample.

The modified Finnegan scoring system is utilized in the Neonatal Intensive Care Unit (NICU) for the assessment and management of neonatal withdrawal. Infants with NAS may necessitate care and intervention in the neonatal intensive care unit (NICU) to alleviate their symptoms. The initial approach to treatment involves non-pharmacological care. Patients should be followed up in a quiet and dimly lit environment and consecutive Finnegan scoring should be performed. Pharmacological treatment should be considered when the Finnegan score is >8 (two consecutive times) or ≥12 [18]. Research indicates that almost 50–80% of patients diagnosed with NAS require pharmaceutical treatment. For patients who are administered pharmaceuticals, the duration of hospital stay is typically around 29 days [28].

According to the latest recommendations of the American Academy of Pediatrics (AAP), first-line pharmacological treatment for newborns exposed to opioids is oral morphine solution, methadone or buprenorphine. Phenobarbital and clonidine are used as second-line treatments for infants not responding to first-line drugs [5]. In the United States, morphine is used in over 80% of infants requiring treatment due to NAS [29]. Currently, the pharmacological management of NAS with morphine demands a lengthy treatment duration and hospitalization [6]. In addition to morphine, phenobarbital is another pharmacological agent used in the management of neonatal abstinence syndrome; however, evidence supporting its efficacy remains limited [30]. Buprenorphine has been suggested as a promising alternative to morphine, with potential advantages including a longer half-life and a wider therapeutic index for respiratory depression [31]. Comparative trials of morphine, methadone, buprenorphine, and phenobarbital have shown variable results, with no clear superiority of any single agent [32,33,34]. In our clinical context, phenobarbital was the initial selection due to the impracticality of titrating morphine sulphate and the frequent supply shortages.

Concerns have been raised regarding the reliability of the Finnegan score, given the presence of symptoms that are not NAS-specific. These symptoms include, but are not limited to, hyperactive Moro reflex, frequent yawning, nasal congestion and sneezing. Furthermore, an infant’s score is based on 21 subjective items, which often leads to variability between scorers. Consequently, a transition from a score-based assessment tool to a function-based tool, from medication management to primarily non-pharmacological care, and from physician-led to family-focused care management has been demonstrated to enhance outcomes for NAS infants. In 2017, Grossman and colleagues developed an approach termed Eat, Sleep, Console (ESC). Subsequent assessment of infants following this programme revealed that those with NAS demonstrated a reduced need for morphine and a concomitant reduction in hospitalisation duration [35]. Notably, a number of studies focusing on quality improvement have indicated a decline in the utilisation of opioid medications for the management of withdrawal symptoms among NAS patients [36].

Substance misuse within families increases the rate of babies being abandoned, particularly in cases where low socioeconomic status and limited education are also present. Following abandonment, the infants require prolonged hospitalization in intensive care due to the involvement of Social Services and the length of time required to complete necessary procedures. In our study, it was discovered that the duration of hospital stay for abandoned infants is notably higher compared to that of babies who were delivered to their families (*p*: 0.02), resulting in a substantial financial burden and overwhelming workload for intensive care units.

A major strength of this study is that it provides regional data on neonatal abstinence syndrome from a setting where evidence remains limited. Most published studies on NAS originate from North America and Western Europe, whereas data from Turkey are scarce and largely restricted to small single-center reports [1,5,37]. Social stigma, limited disclosure, and the absence of systematic perinatal surveillance may contribute to underrecognition of maternal substance use. Therefore, our findings add valuable information to the limited literature and help broaden the understanding of NAS in underrepresented populations.

The study has limitations, including its retrospective and single-centred nature, and the inclusion of both term and premature babies in the sample. Also, our cohort was small and heterogeneous with respect to the substances involved, including opioids, pregabalin, alcohol in combination with other substances, methamphetamine, cannabis, and volatile substances, as well as several cases in which the substance could not be identified. These substances differ in their mechanisms and potential hepatic effects, and the small number of infants per substance class precluded substance-specific subgroup analysis or adjustment. In particular, alcohol-exposed infants were few and all had concomitant polysubstance exposure, so an independent hepatic effect of alcohol could not be isolated. Likewise, although prematurity and perinatal infections (CMV, HIV, syphilis) may influence aminotransferase levels, their low frequency together with the overall sample size did not permit robust multivariable adjustment. A further important limitation is that maternal substance use was ascertained primarily by self-report, with toxicological confirmation available in only two infants; self-report is subject to misclassification, and the specific substance, dose, and timing of exposure may be inaccurate. Although our primary analysis related objectively assessed withdrawal severity to laboratory values and was therefore less dependent on the accuracy of self-reported substance type, residual and unmeasured confounding cannot be excluded.

## 5. Conclusions

In this retrospective, single-centre cohort of neonates with prenatal substance exposure, greater withdrawal severity was associated with higher aminotransferase levels and WBC; however, only the ALT association remained statistically significant after correction for multiple comparisons. Infants placed under state care had significantly longer hospital stays than those discharged to their families.

These findings should be interpreted as preliminary and hypothesis-generating. The small and heterogeneous sample, retrospective design, and lack of systematic toxicological confirmation preclude causal inference. The observed enzyme elevations may reflect substance exposure, withdrawal severity, prematurity, or other perinatal factors that could not be fully disentangled in this cohort.

Future prospective multicenter studies with toxicological confirmation of exposure are needed to clarify the independent relationship between withdrawal severity and hepatic or systemic inflammatory markers. In parallel, the prolonged hospitalization observed among infants entering state care highlights the need for integrated social-support and discharge-planning pathways to reduce the avoidable length of stay.

## Figures and Tables

**Table 1:** Comparison of demographic and clinical features of the patients.

	Finnegan Score < 8 (*n* = 11)	Finnegan Score ≥ 8 (*n* = 14)	*p*
Maternal age ^a^	28.5 ± 6.4	28.8 ± 7.3	0.93
Male sex ^b^	6 (54.5%)	8 (57.1%)	0.6
Gestational week ^a^	36.1 ± 2.3	36.0 ± 2.5	0.9
Gestational weight ^a^	2494 ± 581	2579 ± 387	0.7
Prematurity ^b^	8 (72.7%)	10 (71.4%)	0.65
1.min. APGAR ^c^	7 (3–9)	7 (5–9)	0.6
5.min. APGAR ^c^	8 (6–10)	9 (8–10)	0.27
Resuscitation ^b^	1 (9.1%)	2 (14.3%)	0.6
Need for mechanical ventilation ^b^	3 (27.3%)	5 (35.7%)	0.5
Inotrope support ^b^	1 (9.1%)	3 (21.4%)	0.39
Heroin ^b^	3 (27.3%)	6 (42.9%)	0.35
Abandoned baby ^b^	5 (45.5%)	5 (35.7%)	0.46
Hospitalization day ^a^	27.2 ± 14.4	30.4 ± 17.3	0.31

^a^ mean ± standard deviation, ^b^ number (%), ^c^ median (minimum–maximum).

**Table 2:** Distribution of maternal substances.

Substance	*n* (%)
Heroin	9 (36.0%)
Pregabalin	5 (20.0%)
Alcohol with other substances	3 (12.0%)
Volatile substance, quetiapine	2 (8.0%)
Methamphetamine	1 (4.0%)
Cannabis	1 (4.0%)
Unknown	4 (16.0%)
Total	25 (100%)

**Table 3:** Comparison of laboratory parameters between low- and high-severity Finnegan groups.

Parameter	Finnegan < 8 (*n* = 11)	Finnegan ≥ 8 (*n* = 14)	Mean Difference (95% CI)	Cohen’s d	*p*
WBC (/mm^3^)	11,627 ± 5990	21,176 ± 10,178	9549 (2769, 16,329)	1.11	**0.009**
NLR	2.02 ± 1.60	2.46 ± 1.69	0.44 (−0.93, 1.81)	0.27	0.426
MPV (fL)	9.46 ± 0.34	9.96 ± 0.80	0.50 (0.00, 1.00)	0.78	0.050
PLT (10^3^/mm^3^)	269.7 ± 55.2	320.2 ± 155.1	50.5 (−43.8, 144.7)	0.41	0.298
PCT (%)	0.25 ± 0.05	0.32 ± 0.14	0.07 (−0.01, 0.15)	0.65	**0.041**
CRP (mg/L)	14.28 ± 19.45	12.15 ± 27.45	−2.13 (−21.57, 17.31)	−0.09	0.411
AST (U/L)	56.45 ± 17.23	102.64 ± 52.04	46.19 (14.79, 77.59)	1.13	**0.009**
ALT (U/L)	11.09 ± 2.98	32.50 ± 21.66	21.41 (8.81, 34.01)	1.31	**<0.001**
Creatinine (mg/dL)	0.84 ± 0.13	0.91 ± 0.22	0.07 (−0.08, 0.22)	0.38	0.360

Data are mean ± SD. *p*-values from the Mann–Whitney U test, except MPV and creatinine (Student’s *t*-test). 95% CI denotes the 95% confidence interval of the mean difference. Effect sizes (Cohen’s d) are reported on the means and standard deviations for consistency across all parameters and should be interpreted as approximate for variables with non-normal distributions. Bold indicates *p* < 0.05.

## Data Availability

The data presented in this study are available on request from the corresponding author due to legal restriction.

## Abbreviations

The following abbreviations are used in this manuscript:

NAS	Neonatal Abstinence Syndrome
NICU	Neonatal Intensive Care Unit
AST	Aspartate aminotransferase
ALT	Alanine aminotransferase
CRP	C-reactive protein
WBC	White blood cell
PLT	Platelet
MPV	Mean platelet volume
PCT	Plateletcrit
NLR	Neutrophil-to-lymphocyte ratio
ECHO	Echocardiography
TFUS	Transfontanel ultrasonography
PPV	Positive pressure ventilation
PROM	Premature rupture of membranes
CMV	Cytomegalovirus
HIV	Human immunodeficiency virus
HBsAg	Hepatitis B surface antigen

## References

[1] Haight S.C., Ko J.Y., Tong V.T., Bohm M.K., Callaghan W.M. (2018). Opioid use disorder documented at delivery hospitalization—United States, 1999–2014. MMWR Morb. Mortal. Wkly. Rep..

[2] Keegan J., Parva M., Finnegan M., Gerson A., Belden M. (2010). Addiction in pregnancy. J. Addict Dis..

[3] Lamy S., Laqueille X., Thibaut F. (2014). Consequences of tobacco, cocaine and cannabis consumption during pregnancy on the pregnancy itself, on the newborn and on child development: A review. Encephale.

[4] McGovern R., Bogowicz P., Meader N., Kaner E., Alderson H., Craig D., Geijer-Simpson E., Jackson K., Muir C., Salonen D. (2023). The association between maternal and paternal substance use and child substance use, internalizing and externalizing problems: A systematic review and meta-analysis. Addiction.

[5] Patrick S.W., Barfield W.D., Poindexter B.B. (2020). Committee on Fetus and Newborn, Committee on Substance Use and Prevention. Neonatal Opioid Withdrawal Syndrome. Pediatrics.

[6] Mangat A.K., Schmölzer G.M., Kraft W.K. (2019). Pharmacological and non-pharmacological treatments for the Neonatal Abstinence Syndrome (NAS). Semin. Fetal Neonatal Med..

[7] Shirel T., Hubler C.P., Shah R., Mager A.B., Koch K.L., Sheth D., Uhing M.R., Jones C.W., Field J.J. (2016). Maternal opioid dose is associated with neonatal abstinence syndrome in children born to women with sickle cell disease. Am. J. Hematol..

[8] Jantzie L.L., Maxwell J.R., Newville J.C., Yellowhair T.R., Kitase Y., Madurai N., Ramachandra S., Bakhireva L.N., Northington F.J., Gerner G. (2020). Prenatal opioid exposure: The next neonatal neuroinflammatory disease. Brain Behav. Immun..

[9] Boggess T., Risher W.C. (2022). Clinical and basic research investigations into the long-term effects of prenatal opioid exposure on brain development. J. Neurosci. Res..

[10] Wu P.L., Suen J.L., Yang C.H., Kuo K.C., Yang Y.S.H., Yang S.N. (2021). Enhanced H3K4 Trimethylation in TNF-α Promoter Gene Locus with Cell Apoptosis in the Ventral-Medial Striatum following Opioid Withdrawal of Neonatal Rat Offspring from Morphine-Addicted Mothers. Mediat. Inflamm..

[11] Maguire D., Gröer M. (2016). Neonatal abstinence syndrome and the gastrointestinal tract. Med. Hypotheses.

[12] A Colantonio D., Kyriakopoulou L., Chan M.K., Daly C.H., Brinc D., A Venner A., Pasic M.D., Armbruster D., Adeli K. (2012). Closing the Gaps in Pediatric Laboratory Reference Intervals: A CALIPER Database of 40 Biochemical Markers in a Healthy and Multiethnic Population of Children. Clin. Chem..

[13] Pantea M., Iacob D., Dima M., Prodan M., Belei O., Negrean R.A., Ilie A.C. (2024). Predictive Value of Inflammatory Markers NLR, PLR, APRI, SII, and Liver Function Tests in Systemic Inflammatory Response Syndrome Detection in Full-Term Newborns. Children.

[14] Yin Q., Yin J., Shen L., Zhou Q., Xu W. (2025). The early diagnostic value of neutrophil to lymphocyte ratio and platelet to lymphocyte ratio in neonatal late-onset sepsis. Front. Pediatr..

[15] Bolouki Moghaddam K., Zarkesh M., Kamali A., Dalili S., Heidarzadeh A., Hassanzadeh Rad A. (2015). The Association of Mean Platelet Volume with Intra Ventricular Hemorrhage and Broncho Pulmonary Dysplasia in Preterm Infants. Iran. J. Ped. Hematol. Oncol..

[16] Arshad H., Latif T., Usman M. (2025). Evaluation of Platelet Indices and Sepsis Markers in Neonates with Different Types of Sepsis: Platelet Indices and Sepsis Markers in Neonatal Sepsis. Pak. J. Health Sci..

[17] Tayman C. (2019). Evaluation of Neonatal Abstinence Syndrome (NAS) Frequency and Systemic Inflammatory Response in NAS. Turk. J. Pediatr. Dis..

[18] Raffaeli G., Cavallaro G., Allegaert K., Wildschut E.D., Fumagalli M., Agosti M., Tibboel D., Mosca F. (2017). Neonatal Abstinence Syndrome: Update on Diagnostic and Therapeutic Strategies. Pharmacotherapy.

[19] Lowell A.F., Yatziv T., Peacock-Chambers E., Zayde A., DeCoste C., Suchman N., McMahon T.J. (2022). Reflective functioning in mothers with addictions: Differential relationships involving family history of mental illness and substance use. Front. Psychol..

[20] Gursu R.N., Cetinavci D., Elbe H. (2026). Organ-Specific Histopathological Effects of Prenatal Alcohol Exposure: A Narrative Review. Congenit. Anom..

[21] Gingras J.L., Weese-Mayer D.E., Hume R.F., O’Donnell K.J. (1992). Cocaine and development: Mechanisms of fetal toxicity and neonatal consequences of prenatal cocaine exposure. Early Hum. Dev..

[22] Smith H.S. (2009). Opioid metabolism. Mayo Clin. Proc..

[23] Badakhshan S.N., Ghazizadeh H., Mohammadi-Bajgiran M., Esmaily H., Khorasani M.Y., Bohn M.K., Pashirzad M., Khodabandeh A.K., Zadeh S.G., Alami-Arani I. (2023). Age-specific reference intervals for liver function tests in healthy neonates, infants, and young children in Iran. J. Clin. Lab. Anal..

[24] Victor S., Dickinson H., Turner M.A. (2011). Plasma aminotransferase concentrations in preterm infants. Arch. Dis. Child. Fetal Neonatal Ed..

[25] Ochiai M., Matsushita Y., Inoue H., Kusuda T., Kang D., Ichihara K., Nakashima N., Ihara K., Ohga S., Hara T. (2016). Blood Reference Intervals for Preterm Low-Birth-Weight Infants: A Multicenter Cohort Study in Japan. PLoS ONE.

[26] Ozkan N., Sonmez M.B., Tas Durmus P., Gorgulu Y., Kose Cınar R., Vardar M.E. (2017). Elevated neutrophil lymphocyte ratio in patients with substance use disorders. Eur. Psychiatry.

[27] Soder H.E., Berumen A.M., Gomez K.E., Green C.E., Suchting R., Wardle M.C., Vincent J., Teixeira A.L., Schmitz J.M., Lane S.D. (2020). Elevated Neutrophil to Lymphocyte Ratio in Older Adults with Cocaine Use Disorder as a Marker of Chronic Inflammation. Clin. Psychopharmacol. Neurosci..

[28] Johnson K., Greenough A., Gerada C. (2003). Maternal drug use and length of neonatal unit stay. Addiction.

[29] Patrick S.W., Schumacher R.E., Horbar J.D., Buus-Frank M.E., Edwards E.M., Morrow K.A., Ferrelli K.R., Picarillo A.P., Gupta M., Soll R.F. (2016). Improving Care for Neonatal Abstinence Syndrome. Pediatrics.

[30] Bagley S.M., Wachman E.M., Holland E., Brogly S.B. (2014). Review of the assessment and management of neonatal abstinence syndrome. Addict. Sci. Clin. Pract..

[31] Kraft W.K., Adeniyi-Jones S.C., Ehrlich M.E. (2017). Buprenorphine for the Neonatal Abstinence Syndrome. N. Engl. J. Med..

[32] Davis J.M., Shenberger J., Terrin N., Breeze J.L., Hudak M., Wachman E.M., Marro P., Oliveira E.L., Harvey-Wilkes K., Czynski A. (2018). Comparison of Safety and Efficacy of Methadone vs. Morphine for Treatment of Neonatal Abstinence Syndrome: A Randomized Clinical Trial. JAMA Pediatr..

[33] Nayeri F., Sheikh M., Kalani M., Niknafs P., Shariat M., Dalili H., Dehpour A.-R. (2015). Phenobarbital versus morphine in the management of neonatal abstinence syndrome, a randomized control trial. BMC Pediatr..

[34] Ozcan B. (2021). Neonatal abstinence syndrome in babies of opioid-addicted mothers and follow-up in the neonatal intensive care unit: A single center experience. Ann. Med. Res..

[35] Grossman M.R., Lipshaw M.J., Osborn R.R., Berkwitt A.K. (2018). A Novel Approach to Assessing Infants with Neonatal Abstinence Syndrome. Hosp. Pediatr..

[36] Ryan K., Moyer A., Glait M., Yan K., Dasgupta M., Saudek K., Cabacungan E. (2021). Correlating Scores but Contrasting Outcomes for Eat Sleep Console Versus Modified Finnegan. Hosp. Pediatr..

[37] Elbayiyev S., Çakır U. (2023). Madde Bağımlısı Anne Bebeklerinin Klinik ve Laboratuvar Özellikleri: Tek Merkez Deneyimi. Bağımlılık Derg..

